# Why vaccines matter: understanding the broader health, economic, and child development benefits of routine vaccination

**DOI:** 10.1080/21645515.2019.1708669

**Published:** 2020-01-24

**Authors:** Arindam Nandi, Anita Shet

**Affiliations:** a Center for Disease Dynamics, Economics & Policy, Washington, DC, USA; b Department of International Health, Johns Hopkins Bloomberg School of Public Health, Baltimore, MD, USA

**Keywords:** Routine immunization, child development, broader benefits of vaccines, measles vaccine, cost-effectiveness, benefit-cost

## Abstract

The direct benefits of childhood vaccination in reducing the burden of disease morbidity and mortality in a cost-effective manner are well-established. By preventing episodes of vaccine-preventable diseases, vaccination can also help avert associated out-of-pocket medical expenses, healthcare provider costs, and losses in wages of patients and caregivers. Studies have associated vaccines positively with cognition and school attainment, suggesting benefits of long-term improved economic productivity. New evidence suggests that the measles vaccine may improve immunological memory and prevent co-infections, thereby forming a protective shield against other infections, and consequently improving health, cognition, schooling and productivity outcomes well into the adolescence and adulthood in low-income settings. Systematically documenting these broader health, economic, and child development benefits of vaccines is important from a policy perspective, not only in low and middle-income countries where the burden of vaccine-preventable diseases is high and public resources are constrained, but also in high-income settings where the emergence of vaccine hesitancy poses a threat to benefits gained from reducing vaccine-preventable diseases. In this paper, we provide a brief summary of the recent evidence on the benefits of vaccines, and discuss the policy implications of these findings.

## Introduction

Childhood vaccines save an estimated 2–3 million lives worldwide every year, which has contributed substantially to the reduction in global infant mortality rate from 65 per 1,000 live births in 1990 to 29 in 2018.^1,2^ Vaccines are found to be the most cost-effective approach for reducing childhood disease burden, especially when compared with interventions such as clean water and improved sanitation which can also reduce disease transmission but require expensive and time-consuming infrastructural investment.^3^Cross-national policy efforts such as the World Health Organization’s (WHO) Expanded Programme on Immunization (EPI) of 1974, and the multi-agency Global Alliance for Vaccines and Immunization (Gavi), established in 1999, have supported several countries with research, logistical planning, supply chain management, and financing of national vaccination programs. In recent times, routine vaccination has been supplemented with additional efforts to optimize community coverage. An example is the government of India’s Mission Indradhanush campaign initiated in 2015, that resulted in an increase of full vaccination coverage in target districts by 10 percentage points in just six months.^4^ As a result of these combined in-country and international initiatives, full vaccination rates of children in low-income countries have increased from under 50% to close to 80% during the past two decades.^5^

With such improvements in vaccination rates and reduction in child mortality, future changes in the global child health policy can be envisaged in three broad areas. First, as vaccine coverage improves, and there is increasing protection of both vaccinated and unvaccinated populations through the phenomenon of community immunity, we are likely to see fewer vaccine-preventable diseases in the general population. For example, polio has been eliminated from almost all countries and is at the verge of complete global eradication. However, the growing recognition of the importance of health equity has shown that clusters of susceptible populations within vaccinated societies can preempt disease outbreaks, such as the reemergence of diphtheria infections in Bangladesh and India.^6,7^ Second, the decreasing incidence of vaccine-preventable diseases has diminished the public’s memory of the devastation caused by the diseases, leading to a rise in vaccine hesitancy. Therefore, national programs will have to refocus on maintaining the momentum, although in a world with limited government resources, health policymakers may find it difficult to financially and operationally justify large vaccination programs. Third, the shifting focus from child mortality to morbidity will lead to a greater emphasis on children’s physical, cognitive, and socioemotional development as compared with survival.^8,9^

Due to changing focus from child survival benefits of vaccines to child development benefits, along with greater reliance on multi-criteria decision-making tools, it is more important than ever before to quantify the broader social and individual benefits of vaccination. In this paper, we discuss evidence from a few key studies, and summarize the benefits of childhood vaccines beyond the intended reduction in disease burden and child mortality.

## Economic, equity, and global health benefits of vaccines

Vaccines can have several economic benefits.^3,10^ One of the most discernible benefits is averted medical expenditure. By preventing an episode of the disease through a vaccine, the economic costs of treatment, such as physician fees, drugs and hospitalization expenses, and associated travel costs and wage loss of caregivers could be averted. This is particularly important for low and middle-income countries (LMICs) where a large part of medical expenditure is out-of-pocket. A clear example is the situation in India, where 65% of health expenditure is private, with extreme costs in some cases, which thrusts 51 million people into poverty every year.^2,11,12^ It is estimated that the measles, rotavirus, and pneumococcal conjugate vaccines could help avert $4.6 billion (2016 US$, adjusted for purchasing power parity) in out-of-pocket medical expenses in 41 Gavi-eligible LMICs during 2016–2030.^13^ Vaccines could also reduce the number of people who fall into poverty due to a catastrophic medical expense which is defined as a large proportion (typically, more than 10% to 25%) of household income or expenditure.^12,14-20^

The protection which vaccines provide against the financial risk from a large medical expense can be measured in additional ways. The so-called extended cost-effectiveness (ECEA) studies have estimated large money-metric value of insurance provided by vaccines.^13,14,16,19^ The value of insurance is equivalent to risk premium, which is defined as the amount of money one would be willing to pay in order to avoid the financial uncertainty from a vaccine-preventable disease.^21^ Paying for vaccines, in this context, is akin to paying for a health insurance premium.

Benefit-cost analysis (BCA) studies of vaccines consider a full range benefits as measured by gains in economic productivity. Several alternative BCA methods exist, including a human capital approach which uses the average annual economic contribution of workers, and a friction cost approach which considers productivity lost during the period when a job position remains unfilled due to sickness.^22,23^ Mortality and morbidity risk reduction benefits of vaccines have also been measured in terms the value of statistical life year (VSLY).^24,25^ VSLY is equivalent to the willingness to pay in order to avoid one disability adjusted life year (DALY) from the disease.^26,27^ It is typically measured as a multiple (approximately 2–4 times) of the per capita national income of a country.^28-30^ Newer studies such as those commissioned by the Copenhagen Consensus Center have considered a fixed value of either $1,000 or $5,000 per DALY across all countries and contexts.^29-31^

One of the most comprehensive vaccine BCA studies published recently used the VSLY method and examined the economic benefits of 10 vaccines – for *Haemophilus influenzae* type b, hepatitis B, human papillomavirus, Japanese encephalitis, measles, *Neisseria meningitidis* serogroup A, rotavirus, rubella, *Streptococcus pneumoniae*, and yellow fever – in 73 LMICs.^10^ The authors considered averted medical expenses, transportation costs, and productivity gains in their analysis, and estimated that during 2001–2020, the vaccines together would provide a social and economic value of $820 billion (2010 US$).^10^ During 2011–2020, the rate of return for investment on these vaccines was estimated to be up to 44 times of the initial cost.^32^Routine vaccination has a positive effect on social and health equity among populations. Infectious disease incidence and mortality are often associated with poverty, and exacerbated by lack of access to clean water, sanitation, and basic hygiene among the poor. Routine childhood vaccinations are, thus, estimated to avert the largest burden of diseases, associated medical expenses, and loss in economic productivity in the poorest segments of the society.^13,14,17-19,33,34^ A recent study in 41 Gavi-eligible LMICs found that universal coverage of the measles, rotavirus, and pneumococcal conjugate vaccines would avert a total of 12.6 million cases of catastrophic health expenditure which might have otherwise propelled patients into poverty.^13^ Of those, 75%, 40%, and 22% of cases respectively for the three diseases were from the poorest wealth quintile.^13^

New research shows that vaccines can also tackle global health threats such as antimicrobial resistance (AMR). If left unchecked, AMR-related infections are estimated to result in as many as 10 million deaths per year worldwide by 2050, with an associated global economic cost of US$100 trillion.^35^ Vaccines could prevent infections – either sensitive or resistant – and also reduce the use of antimicrobials, which in turn could slow the growth of AMR.^36-46^

## Child development benefits of vaccines

Persistent or recurrent infections in early life can lead to poor growth and stunting, which in turn can adversely affect adult health, cognitive capacity, and economic productivity.^47-49^ The theoretical basis of the long-term benefits of vaccines is anchored in the widely accepted “fetal origins” hypothesis^50,51^ which links conditions *in utero* and during early childhood with later life outcomes.^48,49,52-66^ Malnutrition, infection, pregnancy and birth complications, and under-stimulation during the first 1000 days of life can have lasting impact on health, cognitive, and economic outcomes well into the old age. In addition to appropriate nutrition and nurturing, health interventions such as routine vaccinations could reduce infectious disease burden in early childhood and thereby help break the intergenerational cycle of poverty, poor health, and low income.

There is a small but growing literature on the potential child development benefits of routine vaccines. The measles vaccine is especially important in this context as episodes of measles could damage protective immune memory for a period of 2–3 years, increasing susceptibility to future measles and non-measles infections.^67-69^ Using sophisticated techniques, scientists have showed that measles infection in children wipes out preexisting antibodies to different pathogens in the months after the infectious episode, leaving them vulnerable to multiple other infections and possible death.^70^A recent longitudinal study of approximately 2,000 children each in Ethiopia, India, and Vietnam has linked measles vaccination at ages 6–18 months of life with 0.1–0.2 higher anthropometric z-scores, 1.7–4.5 percentage points higher scores on standardized cognition tests, and 0.2–0.3 additional schooling grades at ages 7–8 and 11–12 years.^71^ The vaccine has also been associated with 0.2 more schooling grade attainment among South African children and 7.4% higher school enrollment rate among children in Bangladesh.^72,73^

Similar growth, cognition, and schooling benefits have been observed among *Haemophilus influenzae* type B (Hib) vaccinated children in India,^74,75^ and fully vaccinated children in the Philippines.^76^ Another study found that exposure to tetanus vaccination *in utero* increased schooling attainment by 0.3 years for some children in Bangladesh.^77^ At the aggregate level, India’s national vaccination program has been associated with 0.3–0.5 higher height-for-age and weight-for-age z-scores at ages 0–4 years,^78^ and 0.2 additional schooling grades among adults.^79^

## Concluding remarks

Childhood vaccines have numerous positive effects beyond disease prevention. The concept of broader benefits of vaccines which would include cognition, schooling, economic productivity, fertility, and related outcomes was first proposed by a key 2005 article.^80^ During the present decade, researchers have utilized and expanded this framework across several dimensions and country contexts.^76,77,81-85^

A new online database called the Value of Immunization Compendium Evidence (VoICE), created and maintained by the International Vaccine Access Center at the Johns Hopkins University, Bloomberg School of Public Health, now tracks research on the broader benefits of vaccines on health, educational, economic, and equity outcomes worldwide.^86^ The Immunization Economics community of research and practice compiles similar and related information.^87^ Finally, the World Health Organization is developing an approach for systematically measuring the broader benefits, known as the Full Public Health Value Propositions (FPHVP), in the context of LMICs.^88,89^ Regardless of income level, countries around the world are facing a crises in the acceptance of the societal benefits of routine vaccines. Going forward, we hope that these new frameworks will be widely used for child health policy globally.
